# Prevalence of fungal infections using National Health Insurance data from 2009-2013, South Korea

**DOI:** 10.4178/epih/e2014017

**Published:** 2014-09-11

**Authors:** Hee Jung Yoon, Hwa Young Choi, Young Kwon Kim, Yeong Jun Song, Moran Ki

**Affiliations:** 1 Department of Infectious Diseases, Eulji University School of Medicine, Daejeon, Korea; 2 Department of Cancer Control and Policy, Graduate School of Cancer Science and Policy, National Cancer Center, Goyang, Korea; 3 Department of Biomedical Laboratory Science, College of Medical Science, Konyang University, Daejeon, Korea; 4 Department of Preventive Medicine, Eulji University School of Medicine, Daejeon, Korea

**Keywords:** Fungal infections, Mycosis, Prevalence, Health Insurance, Korea

## Abstract

**OBJECTIVES:**

The symptoms of fungal infections vary from minor to severe. According to recent reports, fungal infection prevalence is increasing due to increases in the aging population and in patients with compromised immune systems. However, the total prevalence of fungal infections in South Korea is unknown. We investigated the recent 5-year prevalence of each type of fungal infection in South Korea across age, sex, and regional groups.

**METHODS:**

Nationwide data from the National Health Insurance of South Korea were used. The target population included patients who had received treatment for a fungal infection that was listed as the main disease. For each patient, multiple treatments were counted as one case per year in the data analysis.

**RESULTS:**

The annual prevalence of all-type mycoses increased from 6.9% in 2009 to 7.4% in 2013. Among the specific groups, the prevalence of dermatophytosis was highest (5.2%), followed by that of opportunistic mycoses (1.7%) and superficial mycoses (0.2%). The prevalence of subcutaneous mycoses and generalized mycoses was very low (8 cases per 100,000 persons and 3 cases per 1 million persons, respectively).

**CONCLUSIONS:**

Every year, approximately 7.1% of the Korean population receives treatment for fungal diseases. Cases of opportunistic mycoses are assumed to increase each year. Therefore, further research is needed to understand and monitor the prevalence of mycoses to establish management policies to reduce the burden of fungal diseases.

## INTRODUCTION

Recently, fungal infections have been causing serious problems in aging populations and immune-suppressed patients worldwide. Healthy adults generally have a strong immunity against fungal infections. However, individuals who have a weak immune system such as children, the elderly, those with HIV, and patients who have received a transplant surgery, chemotherapy, or have been taking immunosuppressants for long periods are the most vulnerable to fungal infection [1].

Most fungal infections are not treated well and develops into chronic infections. However, its severity is not considered as high. Worldwide, the infection and mortality rate due to opportunistic mycoses such as *Candida, Aspergillus*, and *Cryptococcus* among patients with weak immunity have been increasing.

According to an analysis based on US death certificate data from 1980 to 1997, the number of deaths due to fungal infection increased 3.4 times from 1,577 to 6,577 [2], and mortality increased from 0.7 to 2.4 per 100,000 persons. However, considering that numerous cases fail to be properly diagnosed with fungal infections among those who are terminally ill, that result is likely underestimated, and more are expected to have died due to a fungal infection. In addition, the fatality rate of invasive mycosis was found to be as high as 22.4% [3], so this infection should be treated carefully.

Marques et al. [4] reported that the prevalence of dermatophytosis worldwide is approximately 20% and that approximately one third of the entire European population is suffering from tinea pedis, also known as athlete’s foot.

Previously reported data on the prevalence of fungal disease in South Korea has come from either a hospital-based retrospective study [5] or case reports on fungal infections [6-10]. Therefore, representative data on the status of fungal infection in South Korea has never been published.

We investigated the epidemiological characteristics of fungal infections in South Korea by sex, age, and the year of data collection from 2009 to 2013 based on disease statistics provided by the Health Insurance Review and Assessment Service (HIRA). These epidemiological data can be utilized as a fundamental resource to establish and evaluate management policies for fungal infections in South Korea.

## MATERIALS AND METHODS

### Data source

Disease statistics from the HIRA were collected based on details provided about medical care expenses that were requested from hospitals and pharmacies. Data on diseases were collected as the patients’ main disease, which have the biggest burden for treatment among the patients’ several diseases. Age was collected as their age at the time of their medical examination, which was calculated using the patients’ date of birth. If one patient have visited the clinic before and after the patient’s birthday, it was counted as two different age of patients.

### Categorization of the targeted disease

Using the disease statistics from HIRA, any patient who requested medical care expenses under the disease code for a fungal infection as the primary diagnosis at least once during the 5-year period (from 2009 to 2013) were eligible for our study. The annual prevalence of fungal infections was calculated by including patients with the disease, who had been treated multiple times were counted as one case per year in the data analysis.

Fungal infection can be categorized into six groups: dermatophytosis (International Classification of Diseases [ICD]-10 code B35), opportunistic mycoses (B37, B44, B45, and B46), superficial mycoses (B36), subcutaneous mycoses (B42, B43, and B47), systemic mycoses (B38, B39, B40, and B41), and other mycoses (B48, and B49) (Appendix 1).

Based on these data, the prevalences by study year, age, sex, and region were calculated by dividing the number of patients with a fungal infection by the number of registered residents according to the National Statistical Office that year. Factors related to these prevalences were analyzed. The regional prevalence was calculated according to the locations of the medical institutions where the treatments had been provided.

## RESULTS

### Prevalence of mycoses

The annual prevalence of all types of mycoses increased from 6.9% in 2009 to 7.4% in 2013. The average annual prevalence was 7.1%. By fungal infection groups, the prevalence of dermatophytosis was highest (5.2%) followed by that of opportunistic mycoses (1.7%). For superficial mycoses, the prevalence was 0.2%. However, subcutaneous mycoses and systemic mycoses showed a very low prevalence of eight per 100,000 persons and three per 1 million persons, respectively (Table 1).

**Figure 1.**
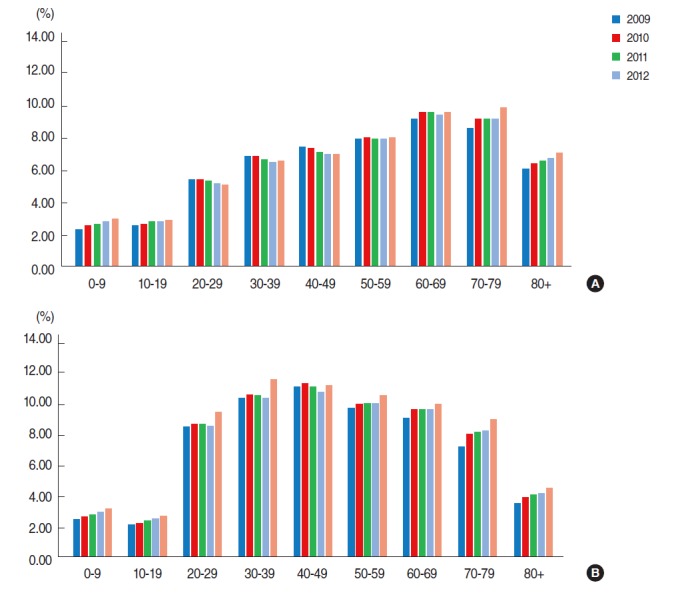
Prevalence of fungal infections by sex (A; male, B; female), age and year, South Korea.

The prevalence among females was 1.3 times higher (8.15%) than that among males (6.09%). Among males, the prevalence continuously increased with increasing age and was highest among those aged 60-69 years but decreased after the 80-89 year old age group. However, the prevalence among females sharply increased from 20-29 years old and was highest among those aged 40-49 years, but then decreased gradually. Overall, increase by year was apparently observed among those under 20 years old and over 70 years old (Figure 1).

**Figure 2.**
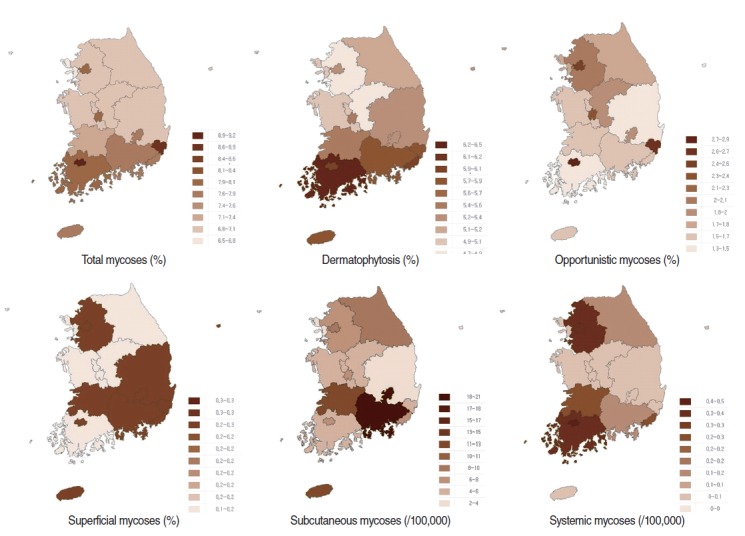
Prevalence of fungal infections by area of hospital and disease group, 2013.

The regional prevalence did not substantially differ across the dataset, was included in our results. The prevalence of fungal infections tended to be high in large cities with big clusters of hospitals (our data were based on the addresses of the medical institutions). For overall mycoses, Gwangju, Ulsan, and Jeonnam Province had the highest prevalence. For dermatophytosis, Jeonnam Province had the highest prevalence, followed by Busan and Gyeongnam Province. Large cities such as Seoul, Daejeon, Ulsan, and Gwangju had a high prevalence of opportunistic mycoses, whereas Busan and Daegu had a relatively low prevalence. The prevalence of superficial mycoses was low in Gangwon Province, Chungbuk and Chungnam Province, and Jeonnam Province compared with other regions. For subcutaneous mycoses, Gyeongnam Province had the highest prevalence, and for systemic mycoses, Seoul, Gyeonggi Province, Gwangju, and Jeonnam had a high prevalence (Figure 2, and Appendix 2).

### Characteristics of prevalence by group of mycoses

#### Dermatophytosis

Dermatophytosis is common skin mycosis, called tinea (B35) with a yearly prevalence of approximately 5.2%. The prevalence among males was higher (5.57%) than that among females was (4.81%). With increasing age, the prevalence also continuously increased and the highest prevalence was among those aged 60-69 years (8.62%), but decreased thereafter (Table 2).

#### Opportunistic mycoses

The prevalence of opportunistic mycoses gradually rose from 1.5% in 2009 to 1.96% in 2013. By sex, the prevalence among females was approximately 15 times higher (3.15%) than that among males (0.22%). By age group, the prevalence was highest among those aged 30-39 years. The average prevalence of candidiasis (B37) was highest at 1.68% over the five study years among all of the types of opportunistic mycoses. As for aspergillosis (B44), zygomycosis (B46), and cryptococcosis (B45), the prevalences per 100,000 persons were 3.880, 0.851, and 0.399, respectively. The average annual number of patients in South Korea was 1,965, 430, and 202, respectively. Candidiasis (B37) differed substantially between males and females (males 0.22%, females 3.14%), and the prevalence was high among those in the 20-29 and 30-39 year old age groups.

For aspergillosis (B44), there was a major difference between the age-specific prevalence; those in the 60-69 and 70-79 year old age groups had the highest prevalence. The prevalence of cryptococcosis (B45) gradually increased with age and was highest among those 70-79 years old. The prevalence of zygomycosis (B46) was high among those younger than 10 years old and those 60 years and older, and it was highest among those 70-79 years old (Table 2).

#### Superficial mycoses

The prevalence of superficial mycoses (B36) was 0.215% in 2009 and 0.206% in 2013, and thus did not change. However, the prevalence was higher among males (0.263%) than that among females (0.155%). No substantial differences were found across age groups, and the prevalence decreased among those 50 years and older (Table 2).

#### Subcutaneous mycoses

For subcutaneous mycoses, the prevalence was 8.155 per 100,000 persons. In 2009 and 2012, the prevalences were 7.4 and 8.3, respectively. Among those with chromomycosis and a phaeomycotic abscess (B43), 2,730 persons per year (5.392 per 100,000 persons) were found. For mycetoma (B47) and sporotrichosis (B42), 1,335 persons per year (2.635 per 100,000 persons) and 65 persons per year (0.129 per 100,000 persons) were found. There was no apparent difference by age and sex group (Table 2).

#### Systemic mycoses

The prevalence of systemic mycoses was 0.2 to 0.3 per 100,000 persons. Among all of the groups with systemic mycoses, the prevalence of coccidioidomycosis (B38) was the highest with a yearly average of 77 persons (0.153 per 100,000 persons). For histoplasmosis (B39), blastomycosis (B40), and paracoccidioidomycosis (B41), we found 27, 19, and 6 persons per year, respectively (0.054, 0.038, and 0.012 per 100,000 persons, respectively.) The prevalence showed an increasing trend by age, but no clear difference by sex was found (Table 2).

#### Other mycoses

The prevalence of other mycoses gradually decreased from 27.9 per 100,000 persons in 2009 to 23.19 per 100,000 persons in 2013. Among females, the prevalence was 28.07 per 100,000 persons, which was higher than that among males (22.04 per 100,000 persons). Moreover, the prevalence was high among those younger than 10 years old and those 50 years and older (Table 2).

## DISCUSSION

According to this research, approximately 7.1% of the entire Korean population receives treatment for fungal disease every year. This yearly rate increased from 6.9% in 2009 to 7.4% in 2013. However, the prevalence for each type of mycosis differed by disease group.

The prevalence of dermatophytosis (tinea) was highest at 5.2%, followed by that of opportunistic mycoses and superficial mycoses at 1.7% and 0.2%, respectively. Since these estimated prevalence rates were calculated by targeting patients who had received hospital treatment for a fungal disease (as the main disease only), the actual prevalence of fungal disease is likely to be higher. In addition, the regional prevalence based on the addresses of the hospitals differed by fungal group. The prevalence was especially high for opportunistic mycoses and systemic mycoses in the big cities, like Seoul. One reason might be that more serious patients such as patients with a suppressed immune system visit hospitals in the big cities due to the fact there is a clustering of major hospitals in these cities.

According to research performed on soldiers in Turkey, the prevalence of tinea pedis was 15.8% in the military population and 4.4% in the civilian population [11]. Besides this previous study, no other national-level study on fungal infections was found.

Dermatophytosis is the most common type of skin infection, and we found that 5.2% of the Korean population receives treatment for it every year. This fungal infection is parasitic and presents in the corneum, hair, or keratin-rich areas such as the fingernails and toenails, and then propagates by feeding on nutriments. Dermatophytosis mostly occurs in people with a normal immune system, and the risk factors are temperature and humidity. Tinea pedis (athlete’s foot) is the most common tinea and accounts for 33%-40% of all tinea cases [12].

In the US, an average of 4,124,038±202,977 yearly visits in the outpatient department was reported for treating dermatophytosis. Among these visits, tinea unguium, tinea corporis, tinea pedis, tinea capitis, and tinea cruris accounted for 23.2%, 20.4%, 18.8%, 15.0%, and 8.4%, respectively [13]. Therefore, dermatophytosis is one of the most common reasons for visiting a dermatologist, and this high prevalence is likely because of the high recurrence rate and fact that it is not typically cured at first incidence [14]. In our study, the prevalence of dermatophytosis was higher among males (5.574%) than that among females (4.81%). This finding is thought to be attributed to the possibility that men tend to participate in sports and/or outside activities more than women do.

When analyzed by age group, superficial mycoses, subcutaneous mycoses, systemic mycoses, and other mycoses were most prevalent among those aged 60-69 and 80-89 years, whereas dermatophytosis and opportunistic mycoses were most prevalent among those aged between 20-39 years. A recent report from South Korea on a group of patients with *Trichophyton mentagrophytes*, which is the second most common type of tinea pedis, found that infection was more prevalent among males than females and was highest among those aged between 30-49 years [5]. They also reported that the number of infected patients decreased since 2005; however, the robustness of these results may be low since it was based on data from only a few hospitals that targeted 6,250 patients over 21 years.

Candidiasis (B37) accounts for the highest prevalence among the opportunistic mycoses and causes various diseases such as mucocutaneous candidiasis and fatal systemic blood stream infection. Candidiasis is one of the common healthcare-associated infection. The extensive use of broad-spectrum antibiotics, anticancer drugs and immunosuppressants, surgery, organ transplantations, the insertion of a prosthetic device or central venous catheter, a high level of nutrition, or the injection of high level nutrients, are some of the common causes of infections in the clinical environment [12,15]. For most fungal diseases, the prevalence does not substantially differ by sex; however, the prevalence of candidiasis (B37) among females was 15 times higher than that among males. One reason for this high occurrence among females might be due to the high prevalence of vaginal candidiasis, especially among females over 30 years of age, according to our research.

The prevalence of aspergillosis (B44) was reported to be increasing because of the increasing number of patients with compromised immunity due to various cancers, organ transplantation, and use of immunosuppressants, but their survival rate has been increasing. Aspergillosis occurs frequently among patients with long-term neutropenia and patients who have undergone stem cell or organ transplantation. In these patients, it usually invades the lungs, paranasal sinus, and central nervous system. In our results, we also found an increasing trend until 2012 and a constant rise in the prevalence of aspergillosis among those over 40 years old. It might be caused by the survival of patients with a weak immunity increases. Therefore, further research about aspergillosis is needed.

Zygomycosis (B46) is caused by saprophytic fungi of the class Zygomycetes, which are primarily opportunists that invade immunocompromised hosts and produce angioinvasive disease [16]. Zygomycosis (also known as mucormycosis) is a very invasive infection that can be fatal among patients with diabetes or a weak immunity; therefore, its mortality rate is high. One case report from South Korea, the mortality rate was 23% (3 died among 13 cases) [17]. Among high-risk patients zygomycoses were reported 3.3 cases and 0.6 cases per 1,000 patients in the patients who had received transplantation and organ transplantation, respectively [12]. Additionally, cases of mucormycosis, which are sudden infections that occur during the administration of voriconazole or preventive antifungal agents for the treatment of aspergillosis, is increasing [12]. In our research, zygomycosis ranked third among all types of opportunistic mycoses and the prevalence was 8-9 cases per 1 million persons. Although this prevalence is not high, there are an increasing number of patients with a weak immunity and an increasing number of cases being prescribed antifungal agents. Therefore, attentive observation is needed.

Cryptococcosis (B45) occurs frequently in patients with an underlying disease, but 20% of these infections have been found to develop in people who do not have a specific underlying disease. The most common type of cryptococcosis is encephalomeningitis [12]. Unfortunately, no data on its prevalence exists in South Korea, but American data from the pre-AIDS period estimated that approximately 40 to 100 cases occur per year [12]. In their data, the prevalence was 3-4 per 1 million persons. Although the prevalence is not high, a slight increasing trend was found annually and with increasing age.

Coccidioidomycosis (B38), which showed the highest prevalence among all types of systemic mycoses, is prevalent in the southwestern areas of the US and is on the rise due to climate changes and shifts in the soil architecture caused by construction developments. In South Korea, 15 cases have been reported since the first report in 1976, and nine of these cases were reported after 2000. Therefore, attention to this imported fungal infection is needed considering its increasing frequency [6].

Histoplasmosis (B39) is prevalent worldwide and is especially prevalent in the Ohio and Mississippi River areas as well as in Central and South America [12]. In South Korea, one case of an AIDS patient who had lived in Guatemala presented with disseminated histoplasmosis capsulati [18]. In our research, it was the second most common type of systemic mycoses, and the prevalence among females was 4 times higher than that among males. Blastomycosis (B40) is also found in the central southern, southeastern, and central western parts of the US as well as Canada, India, the Middle East, South America, and other countries [12]. In South Korea, three cases of pulmonary infection and one case of bone infection with blastomycosis have been reported since 2005 [19]. Two of these cases had previously lived in Tennessee, US [19].

Paracoccidioidomycosis (B41) is prevalent in South America. In Brazil, the prevalence is 3 cases per 100,000 persons with a mortality rate of 2%-23%. In South Korea, there are no records of reported cases; however, based on previous research, it is estimated that approximately six patients become infected per year. For blastomycosis (B40) and paracoccidioidomycosis (B41), the prevalences among males were approximately 3.5 times and 2.5 times higher than that among females, respectively.

The epidemiologic features of fungal infections are not well known despite its high prevalence. According to the McNeil et al. [2] analysis of the mortality rate from infectious diseases in the US, death by fungal infection increased exponentially from 1,557 persons in 1980 to 6,534 persons in 1997, and infection with *Candida, Aspergillus*, or *Cryptococcus* sp. were the principal causes.

It is expected that opportunistic mycosis will likely emerge as the first common fungal infection that will contribute to an increase in medical expenses as the number of patients with weak immunity rise with the extensive use of broad-spectrum antibiotics and the development of medical techniques such as chemotherapy and transplantation that weaken the immune system [20-22]. To reduce the prevalence of opportunistic mycosis infections, measures for hand hygiene, catheter management, and antibiotic management should be implemented. In addition, it is necessary to develop a clinical prediction tool for preventive use against antifungal agents and to educate health care providers about the suitable uses of antifungal agents [23]. In addition, the number of patients with imported fungal infection is expected to increase with increases in air travel. For some imported fungal infections, the mortality rate is high unless there is suitable diagnosis or treatment beforehand. Therefore, caution and careful monitoring through a systematic reporting system is needed.

The first limitation of our study is that the data were based on secondary data, which were insurance claim data collected from main diagnoses from the HIRA. These data do not include cases of patients with a fungal infection who did not receive treatment in a hospital or patients with a main diagnosis of something other than a fungal disease. Second, if a patient received treatment for a fungal infection more than two times in a year, it was counted as one case because recurrence and reinfection are indistinguishable in these data. Thus, our reported prevalence may be underestimated compared to the actual prevalence of fungal infections in South Korea. Third, our data were based on the primary diagnosis by the doctors for insurance purposes; therefore, the accuracy of their diagnoses cannot be guaranteed. Fourth, the regional prevalence was calculated based on the location of the hospitals patients visited, not the patients’ physical addresses. Thus, the regional prevalence may have been influenced by the tendency of patients go visit specific hospitals, especially tertiary hospitals in large cities. Last, the patient’s age provided by HIRA was determined using the date of birth provided at diagnosis, and there were inconsistencies in the prevalence by age due to overlapping data that counted as two cases in a year in some patients. However, the number of patients with overlapping data was 13,765 males (0.78%) and 21,045 females (0.95%) among the 3,808,594 total patient populations with fungal infections. Despite these limitations, our research is meaningful because it provides the first estimated prevalence of fungal disease in South Korea.

In developed countries, national computer networks for reporting fungal infection prevalence, incidence, and mortality are used to monitor epidemiologic trends regularly, but no such system exists in South Korea yet. Based on the information provided above, measures and guidelines focused on reducing fungal infections are needed in South Korea. Furthermore, long-term research and management policies that can also manage new fungal infections are needed.

## Figures and Tables

**Table 1.** Prevalence of fungal infections by disease group and year, South Korea (unit: %)

Mycoses	2009	2010	2011	2012	2013	Annual average
Dermatophytosis	5.1	5.2	5.2	5.1	5.2	5.2
Opportunistic mycoses	1.5	1.6	1.6	1.7	2.0	1.7
Superficial mycoses	0.2	0.2	0.2	0.2	0.2	0.2
Subcutaneous mycoses	0.007	0.008	0.008	0.008	0.008	0.008
Systemic mycoses	0.0002	0.0002	0.0003	0.0003	0.0002	0.0003
Other mycoses	0.028	0.026	0.025	0.023	0.023	0.025
Total	6.9	7.1	7.1	7.1	7.4	7.1

**Table 2.** Prevalence of fungal infections by diseases group, South Korea

	Total mycoses (n, %)		Dermatophytosis	Opportunistic mycoses (/100,000)					Superficial mycoses	Subcutaneous mycoses (/100,000)				Systemic mycoses (/100,000)					Other mycoses (/100,000)		
			(B35, %)	Total	Candidiasis (B37)	Aspergillosis (B44)	Cryptococcosis (B45)	Zygomycosis (B46)	(B36, %)	Total	Sporotrichosis (B42)	Chromomycosis and phaeomycotic abscess (B43)	Mycetoma (B47)	Total	Coccidioidomycosis (B38)	Histoplasmosis (B39)	Blastomycosis (B40)	Paracoccidioidomycosis (B41)	Total	Other mycoses (B48)	Unspecified mycosis (B49)
Annual average	3,602,380	7.115	5.192	1,680	1,675	3.880	0.399	0.851	0.209	8.155	0.129	5.392	2.635	0.257	0.153	0.054	0.038	0.012	25.07	9.499	15.572
Year																					
2009	3,416,637	6.864	5.102	1,512	1,508	3.375	0.356	0.880	0.215	7.416	0.147	4.977	2.292	0.225	0.123	0.064	0.024	0.014	27.911	11.761	16.149
2010	3,582,988	7.093	5.249	1,596	1,591	3.739	0.348	0.996	0.213	8.281	0.141	5.858	2.282	0.234	0.152	0.049	0.02	0.012	26.299	11.195	15.104
2011	3,604,064	7.104	5.216	1,647	1,642	3.924	0.375	0.857	0.208	8.274	0.116	5.202	2.957	0.311	0.179	0.055	0.057	0.02	24.689	10.656	14.034
2012	3,599,619	7.065	5.147	1,681	1,676	4.187	0.434	0.809	0.205	8.416	0.145	5.400	2.872	0.269	0.181	0.051	0.027	0.01	23.259	7.033	16.226
2013	3,808,594	7.447	5.246	1,963	1,957	4.173	0.483	0.714	0.206	8.390	0.094	5.524	2.773	0.246	0.131	0.049	0.063	0.004	23.194	6.850	16.345
Sex																					
Male	7,715,315	6.088	5.574	220	215	4.223	0.380	0.755	0.263	7.966	0.140	5.522	2.304	0.233	0.136	0.021	0.059	0.017	22.043	7.519	14.524
Female	10,296,587	8.148	4.810	3,146	3,141	3.541	0.419	0.946	0.155	8.352	0.117	5.264	2.971	0.282	0.171	0.086	0.017	0.007	28.074	11.452	16.622
Age																					
0-9	636,324	2.691	0.906	1,534	1,532	0.135	0.03	1.205	0.217	6.000	0.038	5.057	0.905	0.275	0.152	0.093	0.013	0.017	28.048	9.159	18.889
10-19	836,645	2.536	1.917	397	397	0.255	0.033	0.218	0.208	6.022	0.039	5.422	0.561	0.091	0.067	0.018	0	0.006	7.828	2.694	5.134
20-29	2,350,705	6.947	3.974	2,654	2,652	0.559	0.077	0.426	0.293	8.517	0.154	6.271	2.092	0.13	0.071	0.056	0.003	0	17.888	7.146	10.742
30-39	3,559,257	8.643	5.431	2,920	2,918	1.425	0.119	0.648	0.258	9.116	0.063	6.012	3.04	0.25	0.177	0.046	0.005	0.022	24.472	10.257	14.215
40-49	4,014,635	9.088	6.614	2,234	2,230	3.429	0.297	0.654	0.205	8.636	0.122	4.957	3.556	0.326	0.199	0.066	0.048	0.014	25.805	11.377	14.428
50-59	3,322,485	8.999	7.545	1,239	1,230	7.619	0.588	0.794	0.176	8.651	0.146	5.003	3.502	0.306	0.187	0.054	0.051	0.014	30.244	11.322	18.922
60-69	2,025,208	9.527	8.622	706	690	13.257	1.308	1.595	0.148	9.470	0.240	5.217	4.013	0.39	0.207	0.056	0.122	0.005	41.319	13.351	27.968
70-79	1,192,731	8.574	7.844	563	545	12.128	1.999	2.919	0.116	9.432	0.381	5.320	3.731	0.359	0.173	0.043	0.122	0.022	41.789	14.27	27.519
80+	248,631	4.797	4.340	355	347	5.769	0.868	1.408	0.067	6.830	0.309	4.090	2.431	0.463	0.212	0.058	0.174	0.019	27.145	8.894	18.251

## Appendix 1. Mycoses disease group (International Classification of Diseases [ICD]-10 code B35-B49)

**Table:**

Group	Diseases
Dermatophytosis	**B35 Dermatophytosis**
	B35.0 Tinea barbae and tinea capitis
	B35.1 Tinea unguium, B35.2 Tinea manuum, B35.3 Tinea pedis, B35.4 Tinea corporis, B35.5 Tinea imbricate, B35.6 Tinea cruris, B35.8 Other dermatophytoses, B35.9 Dermatophytosis, unspecified
Opportunistic mycoses	**B37 Candidiasis**
	B37.0 Candidal stomatitis, B37.1 Pulmonary candidiasis, B37.2 Candidiasis of skin and nail, B37.3 Candidiasis of vulva and vagina, B37.4 Candidiasis of other urogenital sites, B37.5 Candidal meningitis, B37.6 Candidal endocarditis, B37.7 Candidal septicaemia, B37.8 Candidiasis of other sites, B37.9 Candidiasis, unspecified
	**B44 Aspergillosis**
	B44.0 Invasive pulmonary aspergillosis, B44.1 Other pulmonary aspergillosis
	B44.2 Tonsillar aspergillosis, B44.7 Disseminated aspergillosis, B44.8 Other forms of aspergillosis, B44.9 Aspergillosis, unspecified
	**B45 Cryptococcosis**
	B45.0 Pulmonary cryptococcosis, B45.1 Cerebral cryptococcosis, B45.2 Cutaneous cryptococcosis, B45.3 Osseous cryptococcosis, B45.7 Disseminated cryptococcosis, B45.8 Other forms of cryptococcosis, B45.9 Cryptococcosis, unspecified
	**B46 Zygomycosis**
	B46.0 Pulmonary mucormycosis, B46.1 Rhinocerebral mucormycosis, B46.2 Gastro-intestinal mucormycosis, B46.3 Cutaneous mucormycosis, B46.4 Disseminated mucormycosis, B46.5 Mucormycosis, unspecified, B46.8 Other zygomycoses, B46.9 Zygomycosis, unspecified
Superficial mycoses	**B36 Other superficial mycoses**
	B36.0 Pityriasis versicolor
	B36.1 Tinea nigra, B36.2 White piedra, B36.3 Black piedra, B36.8 Other specified superficial mycoses, B36.9 Superficial mycosis, unspecified
Subcutaneous mycoses	**B42 Sporotrichosis**
	B42.0 Pulmonary sporotrichosis, B42.1 Lymphocutaneous sporotrichosis, B42.7 Disseminated sporotrichosis, B42.8 Other forms of sporotrichosis, B42.9 Sporotrichosis, unspecified
	**B43 Chromomycosis and phaeomycotic abscess**
	B43.0 Cutaneous chromomycosis, B43.1 Phaeomycotic brain abscess, B43.2 Subcutaneous phaeomycotic abscess and cyst, B43.8 Other forms of chromomycosis, B43.9 Chromomycosis, unspecified
	**B47 Mycetoma**
	B47.0 Eumycetoma, B47.1 Actinomycetoma, B47.9 Mycetoma, unspecified
Systemic mycoses	**B38 Coccidioidomycosis**
	B38.0 Acute pulmonary coccidioidomycosis, B38.1 Chronic pulmonary coccidioidomycosis, B38.2 Pulmonary coccidioidomycosis, unspecified, B38.3 Cutaneous coccidioidomycosis, B38.4 Coccidioidomycosis meningitis
	B38.7 Disseminated coccidioidomycosis, B38.8 Other forms of coccidioidomycosis, B38.9 Coccidioidomycosis, unspecified
	**B39 Histoplasmosis**
	B39.0 Acute pulmonary histoplasmosis capsulati, B39.1 Chronic pulmonary histoplasmosis capsulati, B39.2 Pulmonary histoplasmosis capsulati, unspecified
	B39.3 Disseminated histoplasmosis capsulati, B39.4 Histoplasmosis capsulati, unspecified, B39.5 Histoplasmosis duboisii, B39.9 Histoplasmosis, unspecified
	**B40 Blastomycosis**
	B40.0 Acute pulmonary blastomycosis, B40.1 Chronic pulmonary blastomycosis
	B40.2 Pulmonary blastomycosis, unspecified, B40.3 Cutaneous blastomycosis
	B40.7 Disseminated blastomycosis, B40.8 Other forms of blastomycosis
	B40.9 Blastomycosis, unspecified
	**B41 Paracoccidioidomycosis**
	B41.0 Pulmonary paracoccidioidomycosis, B41.7 Disseminated paracoccidioidomycosis,B41.8 Other forms of paracoccidioidomycosis
	B41.9 Paracoccidioidomycosis, unspecified
Other mycoses	**B48 Other mycoses, not elsewhere classified**
	B48.0 Lobomycosis, B48.1 Rhinosporidiosis, B48.2 Allescheriasis, B48.3 Geotrichosis, B48.4 Penicillosis, B48.7 Opportunistic mycoses, B48.8 Other specified mycoses
	**B49 Unspecified mycosis**

## Appendix 2. Prevalence of fungal infections by disease group and area of hospital, 2013, South Korea

**Table:**

	Total mycoses (%)	Dermatophytosis (%)	Opportunistic mycoses (%)	Superficial mycoses (%)	Subcutaneous mycoses (/100,000)	Systemic mycoses (/100,000)
Seoul	7.96	5.31	2.40	0.21	9.98	0.39
Busan	7.83	5.96	1.65	0.20	6.60	0.26
Daegu	7.83	5.23	1.86	0.22	20.15	0.08
Incheon	6.58	4.96	1.78	0.18	2.67	0.07
Gwangju	9.16	5.99	2.86	0.29	6.11	0.41
Daejeon	8.07	5.45	2.38	0.19	9.85	0.07
Ulsan	8.85	5.89	2.65	0.29	4.15	0.09
Gyeonggi	7.02	4.71	2.09	0.20	6.48	0.37
Gangwon	7.06	5.12	1.71	0.18	8.17	0.13
Chungbuk	7.02	4.89	1.96	0.15	4.32	0.00
Chungnam^1^	6.83	5.01	1.61	0.19	4.65	0.09
Jeonbuk	7.39	5.44	1.65	0.27	11.21	0.21
Jeonnam	7.95	6.41	1.36	0.16	5.35	0.31
Gyeongbuk	6.86	5.22	1.39	0.24	3.07	0.07
Gyeongnam	7.66	5.78	1.63	0.21	18.72	0.12
Jeju	7.72	5.83	1.66	0.21	12.29	0.00

1 Sejong city is included in Chungnam.

## References

[1] Jain A, Jain S, Rawat S (2010). Emerging fungal infections among children: a review on its clinical manifestations, diagnosis, and prevention. J Pharm Bioallied Sci.

[2] McNeil MM, Nash SL, Hajjeh RA, Phelan MA, Conn LA, Plikaytis BD (2001). Trends in mortality due to invasive mycotic diseases in the United States, 1980-1997. Clin Infect Dis.

[3] Rees JR, Pinner RW, Hajjeh RA, Brandt ME, Reingold AL (1998). The epidemiological features of invasive mycotic infections in the San Francisco Bay area, 1992-1993: results of population-based laboratory active surveillance. Clin Infect Dis.

[4] Marques SA, Robles AM, Tortorano AM, Tuculet MA, Negroni R, Mendes RP (2000). Mycoses associated with AIDS in the Third World. Med Mycol.

[5] Lee WJ, Park KH, Kim MS, Lee SJ, Kim do W, Bang YJ (2014). Decreasing incidence of Trichophyton mentagrophytes in Korea: analysis of 6,250 cases during the last 21-year-period (1992-2012). J Korean Med Sci.

[6] Lee JM, Bae SH, Lee SN, Park KH, Park CK, Yoon HK (2012). A case of disseminated coccidioidomycosis involving the lymph nodes, the skin, and the brain. Korean J Med.

[7] Lee JW, Kim SI, Kim YJ, Kwon JC, Lim YJ, Park MH (2012). A case of coccidioidal meningitis. Infect Chemother.

[8] Kim SW, Oh JY, Kim EJ, Park GM (2009). Pulmonary coccidioidomycosis in immunocompetent patient. Tuberc Respir Dis.

[9] Jhun BW, Kim DM, Park JH, Yoo HS, Shim H, Kim JG (2012). A case of pulmonary blastomycosis mimicking pulmonary tuberculosis. Tuberc Respir Dis.

[10] Chandra H, Chandra S, Sharma A (2012). Histoplasmosis on bone marrow aspirate cytological examination associated with hemophagocytosis and pancytopenia in an AIDS patient. Korean J Hematol.

[11] Senel E, Doğruer Şenel S, Salmanoğlu M (2014). Prevalence of skin diseases in civilian and military population in a Turkish military hospital in the central Black Sea region. J R Army Med Corps.

[12] Korean Society of Infectious Diseases (2014). Infectious diseases.

[13] Panackal AA, Halpern EF, Watson AJ (2009). Cutaneous fungal infections in the United States: Analysis of the National Ambulatory Medical Care Survey (NAMCS) and National Hospital Ambulatory Medical Care Survey (NHAMCS), 1995-2004. Int J Dermatol.

[14] Borman AM, Campbell CK, Fraser M, Johnson EM (2007). Analysis of the dermatophyte species isolated in the British Isles between 1980 and 2005 and review of worldwide dermatophyte trends over the last three decades. Med Mycol.

[15] Kim EC (2011). Current status of healthcare-associated infections in Korea. Hanyang Med Rev.

[16] Anstead GM, Sutton DA, Thompson EH, Weitzman I, Otto RA, Ahuja SK (1999). Disseminated zygomycosis due to Rhizopus schipperae after heatstroke. J Clin Microbiol.

[17] Lee EJ, Chung JW, Choi S, Kim YS, Woo JH (2009). Forefront of diagnosis and treatment of deep-steam mycology in Korea--rhinoorbitocerebral zygomycosis. Nihon Ishinkin Gakkai Zasshi.

[18] Jeong HW, Sohn JW, Kim MJ, Choi JW, Kim CH, Choi SH (2007). Disseminated histoplasmosis and tuberculosis in a patient with HIV infection. Yonsei Med J.

[19] Park KS, Ki CS, Lee NY (2012). Laboratory experience in phenotypic and molecular identification of blastomyces dermatitidis first isolated in Korea. Korean J Clin Microbiol.

[20] Nucci M, Marr KA (2005). Emerging fungal diseases. Clin Infect Dis.

[21] Pfaller MA, Diekema DJ (2004). Rare and emerging opportunistic fungal pathogens: concern for resistance beyond Candida albicans and Aspergillus fumigatus. J Clin Microbiol.

[22] Walsh TJ, Groll A, Hiemenz J, Fleming R, Roilides E, Anaissie E (2004). Infections due to emerging and uncommon medically important fungal pathogens. Clin Microbiol Infect.

[23] Zaoutis TE, Argon J, Chu J, Berlin JA, Walsh TJ, Feudtner C (2005). The epidemiology and attributable outcomes of candidemia in adults and children hospitalized in the United States: a propensity analysis. Clin Infect Dis.

