# Supplementary material for: Prevalence of fungal infections using National Health Insurance data from 2009-2013, South Korea
Source: Epidemiol Health. 2014 Sep 11;36:e2014017. doi: 10.4178/epih/e2014017 (PMC4220602; doi:10.4178/epih/e2014017)
Supplement: Supplementary file 1 [file epih-36-e2014017-supplementary.pdf]

한국의 진균 유병률 - 건강보험자료를 이용한 지난 5 년간 자료분석
Prevalence of fungal infections using National Health Insurance data, from 2009-2013, South Korea

윤희정, 최화영, 김영권, 송영준, 기모란

Hee Jung Yoon1*, Hwa Young Choi2*, Young Kwon Kim3, Yeong Jun Song4, Moran Ki2

[1]Department of Infectious Diseases, Eulji University School of Medicine, Daejeon; [2]Department of Cancer Control and Policy, Graduate School of Cancer Science and Policy, National Cancer Center, Goyang; [3]Department of Biomedical Laboratory Science, College of Medical Science, Konyang University, Daejeon; [4]Department of Preventive Medicine, Eulji University School of Medicine, Daejeon, Korea

Correspondence: Moran Ki

Department of Cancer Control and Policy, Graduat School of Cancer Science and Policy, National Cancer Center, 323 Ilsan-ro, Ilsandong-gu, Goyang 410-769, Korea

Tel: +82-31-920-2736, E-mail: moranki@naver.com

*Yoon & Choi contributed equally to this work as joint first authors.

Abstract

Objectives

진균감염은 증상이 경미한 것부터 심한 것까지 다양하다. 최근에 노령자와 면역저하 환자의 증가로 진균 감염의 증가가 보고 되고 있다. 하지만 진균 전체의 유병률이 보고된 적이 없다. 이 연구는 최근 5 년간 우리나라 인구 전체에서 진균 종류별로 연령별, 성별, 지역별 유병률을 검토하였다.

Methods

전국민을 대상으로 하는 한국의 건강보험 자료를 활용하였다. 2009-2013 년까지 의료기관에서 진균감염을 주상병으로 치료받은 환자를 대상으로 하였으며 여러 번 진료를 받았어도 일년에 1 명의 유병환자로 계산하였다.

Results

진균증 전체의 연도별 유병률은 2009 년 6.9%에서 2013 년 7.4%로 증가하였다. 진균 그룹별로는 피부 진균증의 유병률이 5.2%로 가장 높았고, 다음은 기회감염 진균증으로 1.7%로 나타났다. 표재성 진균증은 0.2%이었다. 하지만 피하진균증은 10 만명당 8 명, 전신성 진균증은 백만명당 3 명의 수준으로 매우 낮은 유병률을 보였다.

Conclusion

우리나라 인구의 약 7.1%가 매년 진균 질환으로 치료를 받고 있는 것으로 나타났다. 특히 기회감염 진균증의 연도별 증가 양상이 관찰되었다. 진균증 유병률에 대한 이해와 관심이 필요하며 이를 토대로 진균 질환 관리 정책 수립이 필요하다.

KEY WORDS: Fungal infections, Mycosis, Prevalence, Health Insurance, Korea

Introduction

최근 전 세계적으로 진균감염이 노령자와 면역저하 환자들에게 심각한 문제를 발생시키고 있다.
건강한 성인의 경우 진균 감염에 대해 강한 면역력을 가지고 있지만, 어린이와 노인, 에이즈 감염자, 이식 수술 환자, 화학 요법제 투여 환자 및 면역억제제의 장기간 사용 환자 등 면역력이 약하거나 약화된 집단이 진균 감염에 가장 취약한 계층으로 알려져 있다 [1].
대부분의 진균감염은 한번 감염되면 잘 치료되지 않고 만성으로 진행하지만 중증도는 높지 않은 질환으로 인식되고 있다. 하지만 면역이 저하된 환자들에서 *Candida, Aspergillus, Cryptococcus* 와 같은 기회감염성 진균에 의한 감염 및 사망률이 전 세계적으로 꾸준한 증가 추세를 보이고 있다. 또한 미국의 1980 년에서 1997 년까지 사망 진단서 자료를 바탕으로 분석한 결과, 진균감염에 의한 사망자수가 1,577 명에서 6,577 명 (100,000 명당 0.7 명에서 2.4 명)으로 3.4 배 증가하였다 [2]. 하지만 이는 중증 환자에서의 진균진단 실패 건까지 고려한다면 과소평가된 결과이며, 실제로는 더 많은 수가 진균감염으로 사망한 것으로 예상하였다. 특히 침습성 진균증의 치명률은 22.4%에 이를 정도로 높아 [3] 주의해야 한다.
국제보건기구(WHO)의 보고에 의하면 전 세계 피부진균증의 유병률이 20%에 근접한 것으로 예측하고 있으며 [4], 유럽 전체 인구 중 1/3 정도가 athlete's foot 으로 불리는 족부백선증을 겪고 있다고 보고하였다.
우리나라의 진균 역학자료로는, 병원기반의 후향적 연구이거나 [5] 침습성 진균감염에 대한 증례보고이다 [6-10]. 따라서 현재까지 우리나라 전체 진균의 유병률을 제시한 보고는 없는 실정이다.
본 논문은 건강보험심사평가원 질병통계를 토대로 최근 5 년간 (2009 년-2013 년)의 진균감염증의 연도별, 성별 연령별 역학적 특성을 제시하고자 한다. 이러한 역학적 자료는 우리나라 진균 감염 관리 정책을 세우고 평가하는데 기초 자료로 활용될 수 있을 것이다.

Materials and Methods

연구 자료

건강보험심사평가원에서 제공하는 질병통계 자료를 이용하였다. 질병통계는 요양기관에서 청구한 요양급여 비용 명세서에 대해 심사 결정한 내용을 기준으로 작성된 것이다. 질병명은 주상병 기준이며, 최종 확정된 질병과는 차이가 날 수 있다. 연령은 환자의 생년월일을 기준으로 계산한 진료 시점의 나이이며, 진료시점이 생일 전후로 2 회 이상이면 유병 환자 2 명으로 중복 집계 될 수 있다.

연구 대상 질병 분류

건강보험심사평가원에서 제공하는 질병통계자료를 통해 2009 년부터 2013 년 최근 5 년동안 한번이라도 주상병에 진균감염 상병코드로 요양급여를 청구한 환자를 대상으로 하였다. 유병환자 기준으로 유병률을 구하기 위하여 같은 상병코드로 2 회 이상 진료를 받은 경우라 하더라도 일년에 1 명의 유병환자로 계산하였다.

진균증은 매우 다양한 질환으로 구성되어 있어서 유사한 형태에 따라 피부 진균증(B35), 기회감염 진균증(B37, B44, B45, B46), 기타 얕은 진균증(B36), 피하 진균증(B42, B43, B47), 전신성 진균증(B38, B39, B40, B41), 기타 진균증(B48, B49) 등 6 개 그룹으로 나누어 분석하였다.(Table 1.) 자세한 그룹별 질병명과 ICD 10th code 는 부록에 첨부하였다. (Appendix 1.)

이 자료들을 기초로 하여 연도별, 연령별, 성별, 지역별 유병률을 구하고 특성을 알아보았다. 각 변수에 대한 유병률은 해당 연도의 진균감염증 환자수를 통계청에서 제공하는 해당 연도 연도말 기준 주민등록인구수로 나누어서 계산하였다. 지역별 유병률은 진료기관의 주소를 기반으로 보았다. 연도별, 성별, 연령별, 지역별 유병률의 차이에 대한 통계적 분석은 모두 유의하였으나 대규모 표본수에 의한 것이어서 결과표에 제시하지는 않았다.

Results

전체 진균증(Total Mycoses)

진균증 전체의 연도별 유병률은 2009 년 6.9%에서 2013 년 7.4%로 증가하였다. 연평균 유병률은 7.1%이었다. 진균 그룹별로는 피부 진균증의 유병률이 5.2%로 가장 높았고, 다음은 기회감염 진균증으로 1.7%로 나타났다. 표재성 진균증은 0.2%이었다. 하지만 피하진균증은 10 만명당 8 명, 전신성 진균증은 백만명당 3 명의 수준으로 매우 낮은 유병률을 보였다. (Table 1.)

성별 유병률은 남자 6.09%, 여자 8.15%로 여성이 1.3 배 높게 나타났다. 남자에서는 연령이 증가하면서 지속적으로 증가하여 60 대에 가장 높고 80 대에는 감소하는 것으로 나타났다. 여자에서는 20 대에 갑자기 증가하여 40 대에 가장 높고 이후 완만히 감소하는 경향을 보인다. 연령별 증가 경향은 20 세 미만, 70 세 이상에서 보다 명확히 관찰되었다 (Figure 1).

지역별 유병률은 연도별 차이가 거의 없어 마지막 연도인 2013 년도만을 대상으로 결과를 제시하였다. 그런데 의료기관 주소를 기반으로 계산하였기에 대부분의 진균 유병률이 큰 병원이 몰려있는 대도시에서 높게 나타나는 경향을 보였다. 전체적으로는 광주와 울산, 전남지역이 높은 유병률을 보였고, 피부진균증은 전남, 부산, 경남의 순으로 높게 나타났다. 기회감염진균증은 서울, 대전, 울산, 광주 등 대도시가 높았으나 부산과 대구는 상대적으로 낮게 나타났다. 표재성 진균증은 강원도, 충북, 충남, 전남이 다른 지역에 비하여 낮게 나타났다. 피하진균증은 경남이 가장 높았고, 전신성 진균증은 서울, 경기와 광주, 전남이 높게 나타났다 (Figure 2). 자세한 지역별, 질병그룹별 유병률은 부록에 첨부하였다 (Appendix 2).

그룹별 진균증 유병률 특성

피부 진균증 (Dermatophytosis)

피부 진균증은 백선증(B35)이 대상질병이며, 매년 5%가 넘는 유병률을 나타내고 있다. 연도별 차이는 없으며 성별로는 남자가 5.57%, 여자가 4.81%로 남자가 높다. 연령이 증가하면서 꾸준히 증가하여 60 대에 가장 높은 유병률(8.62%)을 보이고 이후에 감소하였다 (Table 2).

기회감염 진균증 (Opportunistic mycoses)

기회감염 진균증은 2009 년 1.5%에서 2013 년 1.96%로 점차 증가하고 있다. 성별 유병률은 여자가 3.15%로 남자의 0.22% 비해 약 15 배 높았다. 연령별로는 30 대에 가장 높게 나타났다. 기회 감염 진균증 중 칸디다증(B37)의 유병률이 가장 높아서 최근 5 년간 평균 848,325 명(1.675%) 이었고, 아스페루길루스증(B44), 접합균증(B46), 크립토코쿠스증(B45) 은 각각 인구 10 만명당 3.880, 0.851, 0.399 명 (각각 연간 1,965 명, 430 명, 202 명)으로 나타났다. 칸디다증(B37)은 특히 성별 유병률의 차이가 크게 나타났고 (남 0.22%, 여자 3.14%), 20- 30 대에서 높은 유병률을 보였다.
아스페루길루스증(B44)은 연령별 유병률의 차이가 크게 나타나고 60-70 대에 가장 높은 유병률을 보였다. 크립토코쿠스증(B45)의 연령별 유병률은 점차적으로 증가하다가 70 대에서 가장 높게 나타났다. 접합균증 (B46)의 유병률은 10 세 미만과 60 세 이상에서 높았고 70 대에 가장 높게 나타났다 (Table 2).

표재성 진균증 (Superficial mycoses)

표재성 진균증은 기타 얕은 진균증이 대상 질환이다. 유병률은 2009 년 0.215%에서 2013 년 0.206%로 조금 감소하는 경향을 보인다. 남자는 0.263%, 여자는 0.155%로 남자에서 높고, 연령별로는 큰 차이가 없으나 50 대부터는 감소하는 경향을 보였다 (Table 2).

피하 진균증 (Subcutaneous mycoses)

피하 진균증은 인구 십만 명당 8.155 명 수준의 유병률을 보였다 (2009 년 7.4 명- 2012 년 8.3 명). 피하 진균증 그룹 중에서는 색소진균증 및 갈색진균성 고름집(B43)이 연간 2730 명 정도로 10 만명당 5.392 명 수준이었다. 다음은 균종(B47) (연간 1335 명, 10 만명당 2.635 명), 스포로트리쿰증(B42) (연간 65 명, 10 만명당 0.129 명) 순이었다. 연령별, 성별 차이는 크지 않았다 (Table 2).

전신성 진균증(Systemic mycoses)

전신성 진균증의 유병률은 10 만명당 0.2-0.3 명의 수준으로 나타나고 있다. 전신성 진균증 중에 콕시디오이데스진균증(B38) 의 유병률이 가장 높아 최근 5 년간 연평균 77 명 수준이며(인구 10 만명당 0.153), 히스토플라스마증(B39), 분아균증(B40), 파라콕시디오이데스진균증(B41) 은 각각 연간 27 명, 19 명, 6 명 수준으로 나타났다 (각각 인구 10 만명당 0.054, 0.038, 0.012 명). 연령별로 증가하는 경향을 보이며 성별 차이는 뚜렷하지 않았다 (Table 2).

그 외 진균증 (Others)

그 외 진균증의 유병률은 인구 10 만명당 2009 년 27.9 명에서 2013 년 23.19 명으로 점차 감소하는 것으로 나타났다. 여자의 유병률이 10 만명 28.07 명으로 남자의 22.04 명보다 높았고, 연령별로는 10 세 미만과 50 세 이상에서 높게 나타났다 (Table 2).

Discussion

이 연구 결과 우리나라 인구의 약 7.1%가 매년 진균질환으로 치료를 받고 있는 것으로 나타났다. 연도별로는 2009 년 6.9%에서 2013 년 7.4%로 증가하는 것으로 나타났다. 그러나 각 진균증의 유병 양상은 질환 그룹별로 다르게 나타났다.

이중에 피부진균증(백선증)이 5.2%로 가장 높았으며, 다음은 기회감염 진균증 1.7%, 표재성 진균증 0.2%의 순으로 나타났다. 하지만 본 유병률은 병의원에서 진균질환을 주상병으로 하여 치료받은 환자만을 대상으로 한 것이므로 실제 유병환자수는 이보다 높을 것으로 추정된다. 병의원 주소로 본 지역별 유병률은 진균 그룹에 따라 지역별 차이를 나타내었다. 특히 기회감염진균과 전신성 진균증은 서울 등 대도시에서 높게 나타났는데, 이는 대형병원이 서울 등 대도시에 몰려 있어 면역저하자 같은 중환자들이 더 많기 때문인 것으로 생각된다.

터키에서 군인들을 대상으로 조사한 바에 따르면 피부진균감염이 11%라는 일부 그룹에서의 데이타가 있을 뿐 [11] 다른 나라에서 이렇게 전국민을 대상으로 유병률을 조사한 연구는 찾지 못하였다.

피부진균증은 이 연구결과 인구의 5.2%가 매년 치료를 받는 가장 흔한 피부감염병으로 피부의 각질층, 머리카락, 손발톱과 같은 케라틴에 기생하며 그 영양분으로 번식하는 진균감염이다. 대부분 면역상태가 정상인 사람들에게 발병하며, 온도와 습기가 위험인자이다. 이중 발백선 (무좀)이 가장 흔한 백선으로, 전체 백선의 33-40%를 차지한다 [12]. 미국에서는 피부진균증에 대하여 한해 평균 4,124,038 ± 202,977 번 외래를 방문하였고, 조갑백선, 몸백선증, 무좀, 머리백선, 샅백선이 각각 23.2%, 20.4%, 18.8%, 15.0%, 8.4%를 차지하였다 [13]. 이와 같이 피부진균증은 피부과에 가장 흔히 내원하게 되는 질병이며, 흔히 치료에 잘 듣지 않고 재발이 많아 유병률이 높게 된다 [14]. 피부진균증은 여성보다 남성에서 높았는데 (4.81% vs. 5.574%), 이는 남성이 위험요소인 스포츠나 야외활동을 더 많이 하기 때문으로 생각된다. 연령별로는 표재성 진균증, 피하진균증, 전신성 진균증, 그 외 진균증은 60 대에서 80 대에 가장 높은 유병률이 나타났으나 피부 진균증과 기회감염 진균증은 20 대와 30 대에 높은 유병률이 나타났다. 최근 국내에서 족부백선 중에 2 번째로 흔한 *Trichophyton mentagrophytes* 의 환자군 보고가 있었는데 이 자료에서도 남성이 여성보다 높고 30-40 대에서 높은 것으로 나타났다. 하지만 이 연구에서는 환자수가 2005 년 이후 감소된다고 보고하였으나 몇 개의 병원기반으로 21 년간 6250 명을 대상으로 한 것이어서 발생률이나 유병률이라고 보기 어렵다 [5].

칸디다감염(B37)은 기회감염 진균 중 가장 많은 유병률을 차지하였는데, 점막피부 칸디다증부터 치명적인 전신혈류감염까지 다양한 임상질환을 일으킨다. 최근 광범위 항생제의 사용, 항암제와 면역억제제의 사용, 수술, 장기이식, 인공기구 및 중심정맥 카테터의 삽입, 고농도 영양 및 고농도 영양제 주입 등과 관련되어 의료관련감염의 흔한 원인이 되고 있다. [12, 15] 대부분 진균질환에서 성별 유병률 차이는 크게 없지만, 칸디다(B37) 경우 여성의 유병률이 남성보다 약 15 배 높게 나타났다. 이는 이 연구자료를 근거로 볼 때, 칸디다 질염이 30 대이상의 여성에서 많이 발생하여 유병률이 높은 것으로 생각된다.

아스페루길루스증(B44)은 최근 들어 각종 암 및 장기이식, 면역억제제 사용 등 면역저하환자가 많아지고 이들의 생존율이 높아지면서 증가하는 것으로 보고되고 있다. 특히 장기간 호중구감소증, 조혈모세포 혹은 장기이식환자에서 호발하는데, 폐, 부비동, 중추신경계를 가장 흔하게 침범한다. 이 연구결과에서도 2012 년까지 계속 증가 추세였고, 40 세이후부터 지속적으로 증가추세를 보여 면역저하자들의 생존과 함께 유병률이 상승하는 것으로 추정되며, 추후 이에 대한 요인 분석이 필요할 것으로 보인다.

접합진균(B46)은 Zygomycetes class 의 saprophytic fungi 에 의해 발생하는 것으로 일차적으로 기회감염이고 주로 면역저하자에서 침습성 혈관질환을 일으킨다 [16].

접합진균(mucormycosis 로도 알려져 있음)은 매우 침습적이고 당뇨환자나 면역저하 환자에서 치명적인 중증감염을 일으키고 사망률이 높은 진균이다. 국내의 한 증례 보고에 의하면 치명률이 23%에 이른다고 하였다 (13 명 중에서 3 명 사망) [17]. 그러나 우리나라의 정확한 발생률은 모른다 [12]. 고위험군 환자 중 이식환자 1,000 명에서 3.3 건, 장기이식환자 1,000 명에서 0.6 건이 보고되었고, 각각 진균감염의 7%, 2%를 차지하였다. 또한 아스페루길루스 치료를 위해 voriconazole 을 투여하거나 예방적 항진균제 투여 중 돌발감염으로 발생하는 털곰팡이증 증례가 증가하고 있다 [12]. 이 연구에서는 기회진균 중 세번째로 백만명당 8-9 명의 발생수준을 보여 그리 높지 않으나 면역저하자 증가 및 항진균제 처방사례가 늘고 있는 만큼 주의해서 관찰해보아야 할 질환이다.

크립토코쿠스증(B45)은 기저질환이 있는 환자에서 많이 발생하지만, 20%는 특이질환이 없는 사람에게서도 발생하며 뇌수막염이 형태가 가장 흔하다 [12]. 국내에서는 정확한 자료가 없지만 에이즈 이전 시대의 미국자료에 근거하여 추정하면 연간 약 40 건-100 건 정도가 발생할 것으로 추정한다. [12] 이 연구자료에서는 백만명당 3-4 명의 수준으로 유병률은 높지 않으나 해마다 약간씩 증가 추세를 보이고 있고 연령이 높아질수록 증가하였다.

전신성 진균증 중 가장 높은 유병률을 보인 콕시디오이데스 진균증(B38)은 미국 서남부 지역에서 유행하며, 최근 기후 변화 및 개발 공사로 인한 토양의 변화 때문에 증가추세이다. 국내에서는 1976 년 첫 보고 이후 15 예가 보고 되었고, 그 중 9 예는 2000 년 이후 보고되어 최근 빈도가 높아지고 있는 해외 유입성 진균으로 주의를 요한다 [6]. 히스토플라스마증(B39)은 전세계적인 분포를 보이며 특히 오하이오와 미시시피강 지역과 중남미 지역이 유행지역이다 [12]. 우리나라에서는 2007 년 과테말라에서 거주했던 AIDS 환자에서 파종성 히스토플라스마증이 보고된 1 예가 있다 [18]. 이 연구에서는 두번째로 많이 발견되는 전신성 진균이며 여성이 남성에 비해 약 4 배 높은 것으로 나타났다. 분아균증(B40)은 미국 중남부, 동남부, 중서부 및 캐나다, 인도, 중동, 남미 등에서도 발병이 있다 [12]. 우리나라에서는 2005 년 이래 폐감염 3 례, 골감염 1 례가 보고되었고, 이중 2 례는 미국 테네시 지역에 거주한 경험이 있었다 [19]. 파라콕시디오이데스진균증(B41)은 남미에 만연하는 질병으로 브라질의 경우 인구 10 만명당 3 명이 발병하며 치사율은 2-23%이고 우리나라는 아직 보고된

증례는 없었으나, 연구 결과 1 년에 6 명 정도의 환자가 발생하는 것으로 보인다. 분아균증(B40)과 파라콕시디오이데스진균증(B41)의 경우 남성이 여성에 비해 약 3.5 배와 2.5 배 높게 나타났다.

진균감염증은 유병률이 높음에도 불구하고 역학적 특성이 잘 알려지지 않고 있다. McNeil 등은 [2] 미국에서 감염병에 의한 사망률을 분석한 결과 진균에 의한 사망이 1980 년도에 1,557 명에서 1997 년 6,534 명으로 기하급수적으로 증가한 것을 발견하였고, *Candida*, *Aspergillus*, *Cryptococcus* sp. 감염이 주된 요인이라고 발표하였다. 광범위 항생제의 사용, 항암요법, 이식 등의 의료기술의 발달로 면역저하자는 증가추세이기 때문에, 조만간 기회진균감염 증이 첫번째 진균감염으로 대두되어 의료비증가에 기여할 것이다 [20-22]. 기회진균감염을 줄이기 위해서는 손위생, 카테터 관리, 항생제 관리 등을 잘 시행해야 하며, 항진균제의 예방적 사용기준을 알 수 있는 임상 예측지표 등의 개발, 적절한 진균제 사용에 대한 의료인의 교육이 필요하겠다 [23]. 또한, 비행이동인구가 증가하면서 해외 유입진균환자도 증가할 것으로 생각된다. 일부 해외 유입진균 감염은 진단이나 적절한 치료가 선행되지 않을 경우 치사율이 높아 주의가 필요하며, 체계적 보고 관리를 통해 모니터링이 필요할 것으로 생각된다.

이 연구의 제한점은 건강보험심사평가원에서 주상병 기준 보험청구를 한 자료를 대상으로 정리하여 제공하는 2 차 자료를 활용하였다는데 있다. 그렇기 때문에 진균감염이 있다 하더라도 병원에서 치료를 받지 않은 사람이거나, 다른 질환을 주상병으로 하면서 진균질환은 부상병으로 치료를 받은 사람들 역시 포함되지 않는다. 또한 한 사람이 일년에 2 회 이상 같은 진균에 감염되어 치료받았다 하더라도 이 자료에서는 재발과 재감염을 구분할 수 없어 한번의 발생으로 계산하였다. 따라서 이 자료는 우리나라의 실제 진균 유병률보다 낮게 추정된 것일 수 있다. 또한 이 자료는 의사가 보험 청구할 때 사용한 진단명을 기준으로 하였으나 진단의 정확도는 알 수 없는 단점이 있다. 그리고 지역별 유병률은 환자의 주소가 아니라 환자가 이용한 병의원의 주소를 기준으로 한 것이어서 질병에 따라 특정 병의원으로 가게 되거나 대도시의 3 차병원으로 가는 경향이 있을 수 있어 이를 감안하여 평가해야한다. 그리고 건강보험심사평가원에서 제공하는 연령별 환자수 기준이 수진자 생년원일을 기준으로 한 것이어서 연령별 유병률에서 일부 환자가 1 년에 2 회 중복 집계되는 문제점이 발생하였다. 그러나 전체 진균감염 환자수 3,808,594 명 중 연령이 중복 집계된 환자수는 남자 13,765 명(0.87%) 여자 21,045 명(0.95%)으로 연령별 유병률에 큰 영향을 주지는 않았을 것으로 보인다.

이 연구는 이러한 제한점에도 불구하고 전국민을 대상으로 진균질환 전체의 유병률을 국내 최초로 검토하여 제시하였다는데 그 의의가 있다.

선진국은 전국 전산망을 구축하여 국가전체의 진균 유병률, 발생률, 사망률 등을 보고하고 역학 추세를 주기적으로 모니터링하고 있으나 국내에서는 아직 그러한 시스템이 없다. 위 내용들을 토대로 국내에서도 향후 발생 가능한 진균감염 사례를 미리 예측 할 수 있는 선행적인 대처 방안 및 가이드라인을 준비하는 것이 필요하다. 또한 장기적으로 새로운 진균 감염에 대비한 조사연구 및 관리 정책이 필요하다고 판단된다.

REFERENCES

1.Jain A, Jain S, Rawat S. Emerging fungal infections among children: A review on its clinical manifestations, diagnosis, and prevention. J Pharm Bioallied Sci 2010;2:314-320.

2.McNeil MM, Nash SL, Hajjeh RA, Phelan MA, Conn LA, Plikaytis BD, et al. Trends in mortality due to invasive mycotic diseases in the United States, 1980-1997. Clin Infect Dis 2001;33:641-647.

3.Rees JR, Pinner RW, Hajjeh RA, Brandt ME, Reingold AL. The epidemiological features of invasive mycotic infections in the San Francisco Bay area, 1992-1993: results of population-based laboratory active surveillance. Clin Infect Dis 1998;27:1138-1147.

4.Marques SA, Robles AM, Tortorano AM, Tuculet MA, Negroni R, Mendes RP. Mycoses associated with AIDS in the Third World. Med Mycol 2000;38 Suppl 1:269-279.

5.Lee WJ, Park KH, Kim MS, Lee SJ, Kim do W, Bang YJ, et al. Decreasing incidence of Trichophyton mentagrophytes in Korea: analysis of 6,250 cases during the last 21-year-period (1992-2012). J Korean Med Sci 2014;29:272-276.

6.Lee JM, Bae SH, Lee SN, Park KH, Park CK, Yoon HK, et al. A Case of Disseminated Coccidioidomycosis Involving the Lymph Nodes, the Skin, and the Brain. Korean J Med. 2012;82:734-738.

7.Lee JW, Kim SI, Kim YJ, Kwon J-C, Lim YJ, Park MH, et al. A Case of Coccidioidal Meningitis. Infection & Chemotherapy 2012;44:75-79.

8.Kim SW, Oh JY, Kim E-J, Park GM. Pulmonary Coccidioidomycosis in Immunocompetent Patient. Tuberculosis and Respiratory Diseases 2009;66:220-224.

9.Jhun BW, Kim DM, Park JH, Yoo HS, Shim H, Kim JG, et al. A Case of Pulmonary Blastomycosis Mimicking Pulmonary Tuberculosis. Tuberculosis and Respiratory Diseases 2012;72:77-81.

10.Chandra H, Chandra S, Sharma A. Histoplasmosis on bone marrow aspirate cytological examination associated with hemophagocytosis and pancytopenia in an AIDS patient. Korean J Hematol 2012;47:77-79.
11.Senel E, Dogruer Senel S, Salmanoglu M. Prevalence of skin diseases in civilian and military population in a Turkish military hospital in the central Black Sea region. J R Army Med Corps 2014
12.Infectious Diseases. 2nd ed. Seoul: Koonja publishing; 2014
13.Panackal AA, Halpern EF, Watson AJ. Cutaneous fungal infections in the United States: Analysis of the National Ambulatory Medical Care Survey (NAMCS) and National Hospital Ambulatory Medical Care Survey (NHAMCS), 1995-2004. Int J Dermatol 2009;48:704-712.
14.Borman AM, Campbell CK, Fraser M, Johnson EM. Analysis of the dermatophyte species isolated in the British Isles between 1980 and 2005 and review of worldwide dermatophyte trends over the last three decades. Med Mycol 2007;45:131-141.
15.Kim E. Current Status of Healthcare-associated Infections in Korea. Hanyang Medical Reviews 2011;31:135-140.
16.Anstead GM, Sutton DA, Thompson EH, Weitzman I, Otto RA, Ahuja SK. Disseminated zygomycosis due to Rhizopus schipperae after heatstroke. J Clin Microbiol 1999;37:2656-2662.
17.Lee EJ, Chung JW, Choi S, Kim YS, Woo JH. Forefront of diagnosis and treatment of deep-steam mycology in Korea--rhinoorbitocerebral zygomycosis. Nihon Ishinkin Gakkai Zasshi 2009;50:229-233.
18.Jeong HW, Sohn JW, Kim MJ, Choi JW, Kim CH, Choi SH, et al. Disseminated histoplasmosis and tuberculosis in a patient with HIV infection. Yonsei Med J 2007;48:531-534.
19.Park KS, Ki CS, NY L. Laboratory Experience in Phenotypic and Molecular Identification of Blastomyces dermatitidis First Isolated in Korea. Korean J Clin Microbiol. 2012;15:114-116.
20.Nucci M, Marr KA. Emerging fungal diseases. Clin Infect Dis 2005;41:521-526.
21.Pfaller MA, Diekema DJ. Rare and emerging opportunistic fungal pathogens: concern for resistance beyond Candida albicans and Aspergillus fumigatus. J Clin Microbiol 2004;42:4419-4431.
22.Walsh TJ, Groll A, Hiemenz J, Fleming R, Roilides E, Anaissie E. Infections due to emerging and uncommon medically important fungal pathogens. Clin Microbiol Infect 2004;10 Suppl 1:48-66.
23.Zaoutis TE, Argon J, Chu J, Berlin JA, Walsh TJ, Feudtner C. The epidemiology and attributable outcomes of candidemia in adults and children hospitalized in the United States: a propensity analysis. Clin Infect Dis 2005;41:1232-1239.
